# 

**Published:** 1886-10-16

**Authors:** 


### The Hospital.

An Institution, Family, and Parochial Journal of Hospitals, Asylums, and all Agencies or the Care of the Sick, Criticism and News.

SATURDAY, OCTOBER 16TH, 1886.

### NOTICE TO CORRESPONDENTS.

All correspondence must be written on one side of the paper only.

We cannot undertake to return MSS. not used.

**Communications,** whether intended for insertion or for private information, must be authenticated by the names and addresses of the writers, not necessarily for publication.

**Local papers** containing reports or news paragraphs **should be marked.**

It is especially requested that early intelligence of **local events of interest,** or which it may be thought desirable to bring under notice, may be sent direct to the Editor.

Communications respecting editorial matters should be addressed to the Editor, Norfolk House, Norfolk Street, Strand, London, W.C.

"VICTORIA."—Please send us your present address to Norfolk House, Norfolk Street, W.C.

### "The Hospital" Prize Competitions.

We offer for competition among our readers money prizes, classified under the subjoined heads. We are anxious that this feature of The Hospital shall prove of interest to all classes of readers, and the grouping of the competitions has been arranged solely with this end in view.

All competitors who send in papers must strictly abide by the following

### Conditions.

I. Every paper sent in must be clearly written, and one side only must be used. All competitors must mark at the head of their papers the number of their competition.

II. Each paper must be signed by the author with his or her real name and address. A *nom de plume* may be added, if the writer does not desire to be referred to by us by his real name. In the case of all prize-winners, however, the real name and address will be published.

III. All communications relating to prize competitions should be addressed to "The Prize Editor, *The Hospital* Office, Norfolk House, Norfolk Street, Strand, W.C." Competitors are requested not to include in letters, etc., to the Prize Editor communications on matters of other business. All letters must be fully prepaid.

IV. The Prize Editor of *The Hospital* is the sole judge of the competitions, and his decision is in all cases final and without appeal.

**V. All competitions must be received at "The Hospital" Office by the dates stated, failing which they cannot be dealt with. Every Reply must be accompanied by the Prize Competition "Coupon" which appears on another page.**

VI. The Prize Editor reserves the right to publish any paper sent in, whether it receives a prize or not. He does not hold himself responsible for any MSS. sent, nor can he undertake to return rejected competitions.

VII. No competition must exceed two columns in length, except the Story competition, which must not exceed six columns.

### The One Guinea Prize.

A prize of *One Guinea* will be offered every fortnight to all readers of *The Hospital* for the best *Story* or narrative based upon hospital, asylum, or student life, or upon any incident connected with the professions of medicine or nursing. The Prize Competition will be published in *The Hospital.*

Competitions must be received by Saturday, 30th October. Envelopes to be marked in left-hand corner, "Story."

### The Half-Guinea Prize.

A prize of *Half-a-Guinea* is offered fortnightly to in and out-patients for the best *Anecdote* or *Personal Reminiscence* of any interesting circumstance which may have come under their observation while at or attending any hospital.

Competitions must be received by Saturday, 30th October. Envelopes to be marked in left-hand corner, "Anecdote" or "Reminiscence."

### The Five Shilling Prize.

A Prize of *Five Shillings* is offered fortnightly to all readers of *The Hospital* for the best *Item of News* suitable for publication in this Journal.

Competitions must be received by Saturday, 30th October. Envelopes to be marked in left-hand corner, "News."

### The Nine Pounds Quarterly Prizes.

Prizes of *Five, Three,* and *One Pound* will be awarded on 2nd January, 1887, to the three persons who shall have obtained during the months of October, November, and December, 1886, the largest number of subscribers to *The Hospital.* Order Forms, for subscribers to fill up and sign, may be obtained on application to the publishers, 30 and 32, Sardinia Street, Lincoln's Inn Fields, London, W.C. The Prize Editor must be informed by competitors from time to time of the names and addresses of new subscribers they procure. Names will be received up to and including Saturday, 23rd December, 1886.

### "The Hospital" Charity Box.

Our "Charity Box" is intended to afford a little practical help to the hospitals. Each week we propose a word for "anatomical dissection," and the first 150 competitors who extract the largest number of words from it receive the following prizes:—

| | | |
|---|---|---|
| 1st | ... ... | One Guinea. |
| 2nd | ... ... | Half a Guinea. |
| 3rd | ... ... | Five Shillings. |

The above Prizes will be repeated for every additional 150 competitors who may enter each week.

### Third Competition.

Compose the largest number of words you can from the name—

JOHN HUNTER.

*Observe:* Proper names, abbreviations, foreign and obsolete words, and repetitions are barred.

Write only on one side of the paper, and total your words.

Append your real name and address.

All replies, addressed "Charity Box," The Hospital, Norfolk House, Norfolk Street, W.C., must arrive by Friday, October 22nd. Results will appear Saturday, October 30th.

*Every Competitor is required to cut out and enclose with his list the "Charity Box" Coupon* (see advertisement pages), *and a shilling entrance fee* (postal orders only).

The proceeds resulting from each "Charity Box" competition will be funded until the end of December 1886. The total sum available for distribution, and the names of the hospitals selected for donations, will appear on Saturday, January 8, 1887. The distribution of the proceeds will be at the Editor's sole discretion.

### Results of First Competition.

The "Abernethy" Competition has resulted as follows:—

| | Score. | |
|---|---|---|
| Mr. Wallace A. Smith, 7, Crane Street, Chester ... ... | 162 | 1st Prize. |
| Miss S. M. Wheeler, 9, Rupert Road, Bedford Park, W. ... | 154 | 2nd Prize a "tie." |
| Mr. E. Bruce, 15, Holbeck Road, Brixton, S.W. ... ... ... | 154 | 2nd Prize a "tie." |
| Mrs. L. Wetherfield, 13, Highbury Grove, N. ... ... | 145 | 3rd Prize. |

The Competition was very closely contested. The reduced totals of the above, and of some of the unsuccessful competitors, are due to the fact that inadmissible words have been introduced, such as purely Latin terms, abbreviated forms, archaic words, prefixes and affixes, etc. Different "spellings" only (and *not* variations of meaning of similarly spelt words) are allowed to count. This is what we mean by "repetitions barred."

We have forwarded the prizes to the winners; the second being a "tie," that prize has been divided.

### THE CHILDREN'S CORNER.

"Dear Mr. Editor," writes a correspondent, who wishes to be known as "Little Mary," "I am so glad you have not forgotten us children, for I am sure the 'Corner' which you have reserved for us will lighten the heart of many a little convalescent. I am quite well now, but back in the summer I was in a hospital for nearly six weeks;—how I wish your nice paper had been published then." "Little Mary's" answer is typical of many more which I have received, and I need scarcely say how gratifying these little epistles have been to me.

### OUR PASTIMES.

First Quarterly Prize Competition—

| | |
|---|---|
| Began ... ... ... | 2nd Oct., 1886 |
| Ends ... ... ... | 25th Dec., 1886 |

| | |
|---|---|
| 1st ... ... ... ... | Thirty shillings. |
| 2nd ... ... ... ... | Twenty " |
| 3rd ... ... ... ... | Ten " |
| 4th ... ... ... ... | Five " |

will be awarded to the four competitors who shall have sent in, durirg the thirteen weeks, the largest number of correct answers to our pastimes.

THIRD WEEK.

No. 10. What is that which, though formed long ago, we make every day, and which, while we none of us wish to keep it, yet never desire to give it away?

No. 11. Who can render the following into an English sentence?

Voluntas sum currit
Contra homo dic pax.

No. 12. I am of eleven letters; my 6, 4, 8, 7, is a vessel; my 7, 4, 8, 10, 11, is a bottle; my 7, 3, 1, 6, is what all children are; my 2, 3, 10, 11, 9, 4, is what everybody longs to possess, and my whole is what everybody longs to read.

No. 13. Who can read the following sentence?

Ourselves, Sir, ourselves
rate standing
you my

No. 14. What is that word of one syllable, which, if you deprive of a letter, at once becomes a word of two syllables?

No. 15. There is a pole twelve feet high. A snail climbs up it one foot a day, but falls down six inches each night. How long will he be in arriving at the top?

Results of First Competition.

No. 1. My little friends have discovered four or five words from which, if you take away one letter, only "one" remains. "N-one" was the word I intended, but "on(c)e," "(t)one," etc., will answer the conditions, and I therefore accept their answers as right.

No. 2. The maiden would probably reply, I *can, Sir* (Cancer).

No. 3. The sum in "puzzle arithmetic" has beaten all my little correspondents, though at this I am scarcely surprised. Master Fred. Moore, who dates from a Grammar School, boldly asserts that

$$\frac{100+50}{0}=150\ (!)$$

Probably, before he leaves school, he will learn that such an expression signifies infinity, but "puzzle arithmetic" does not mean a mathematical performance of the usual kind. Let us take the question:

100+50 in *Roman* figures=C plus L. Now, C plus L *divided by* O=COL. Again, two-thirds of t-e-n=E N. And lastly, we are told that "SO" my question ends. Hence, the answer COLENSO, the author of the well-known arithmetic!

No. 4. This word of strange sexes is Heroine, which gives us successively, He, her, hero, heroine.

The answers which I have received from my little readers show a considerable amount of intelligence, and for the most part are characterised by exceeding neatness. The replies from "Mabel," "Alsie," and "Ubique," were little models of penmanship.

All right—None.
Three right—Little Mary, Alsie, J. Foster, Ethel.
Two right—Fred. Moore, Ubique, Retsibsi, C. Tynget, Diosota, Bismarck, Katie King, and Nonsuch.
One right—George Collins.

Answers, having the actual name and address (to which a pseudonym may be added for publication), must have "The Children's" Coupon attached (see advertisement pages), addressed to the "Puzzle Editor, The Hospital, Norfolk House, Norfolk Street, Strand, W.C.," not later than Thursday, 21st October. Results will appear in our issue of the 30th inst.

### TERMS OF SUBSCRIPTION.

Subscribers to The Hospital residing in London and the Suburbs will receive their Copies by the first post on Friday Morning. Copies for Country Subscribers are forwarded by the Day Mail on Fridays. Terms, including postage, payable in advance:

| | Weekly Parts. | Monthly Parts. |
|---|---|---|
| Twelve months ........ | 0 6 6 .... | 0 7 6 |
| Six " ........ | 0 3 3 .... | 0 3 9 |
| Three " ........ | 0 1 8 .... | 0 1 11 |

Post Office Orders should be made payable at the General Post Office, London, E.C., to Whiting and Co., 30 and 32, Sardinia Street, Lincoln's Inn Fields, W.C. Cheques to be crossed "London and Joint Stock Bank, Limited," Chancery Lane Branch.

**Cases for Binding** the weekly or monthly parts of The Hospital in yearly vols. may be ordered of the Publishers.

### BUSINESS COMMUNICATIONS AND ADVERTISEMENTS.

All communications concerning business matters, non-delivery of The Hospital, etc., should be addressed to the Manager, at the Publishing Offices, 30 and 32, Sardinia Street, Lincoln's Inn Fields, London, W.C.

**Cheap Advertisements** of the "Wanted" class, including those for situations, of Twenty-four Words or under, are charged 1*s*. an insertion, or three insertions for 2*s*. 6*d*., but must be paid for in advance. Each additional line of Eight Words is 4*d*.

**Employers requiring assistants** are charged for one insertion of Twenty-four Words or under, 2*s*. 6*d*., or 6*s*. for three insertions. Each additional line of Eight Words is 1*s*.

**Advertisements** for insertion in the weekly issue of The Hospital **must be delivered** at the Publishing Offices **not later than 11 a.m. on Wednesdays.**

Cheques and Post Office Orders to be made payable to Messrs. Whiting & Co. Subscribers and Advertisers are requested **not to send stamps.**

